# Impact of double reading on NI-RADS diagnostic accuracy in reporting oral squamous cell carcinoma surveillance imaging – a single-center study

**DOI:** 10.1259/dmfr.20210168

**Published:** 2022-01-01

**Authors:** Fabian Henry Jürgen Elsholtz, Sa-Ra Ro, Seyd Shnayien, Patrick Dinkelborg, Bernd Hamm, Lars-Arne Schaafs

**Affiliations:** 1 Department of Radiology, Charité – Universitätsmedizin Berlin, corporate member of Freie Universität Berlin, Humboldt-Universität zu Berlin, and Berlin Institute of Health, Campus Benjamin Franklin, Berlin, Germany; 2 Department of Oral and Maxillofacial Surgery, Charité – Universitätsmedizin Berlin, corporate member of Freie Universität Berlin, Humboldt-Universität zu Berlin, and Berlin Institute of Health, Campus Benjamin Franklin, Berlin, Germany

**Keywords:** Head and neck cancer, Squamous cell carcinoma, Surveillance, NI-RADS, Education

## Abstract

**Objectives:**

The Neck Imaging Reporting and Data System (NI-RADS) is an increasingly utilized risk stratification tool for imaging surveillance after treatment for head and neck cancer. This study aims to measure the impact of supervision by subspecialized radiologists on diagnostic accuracy of NI-RADS when initial reading is performed by residents.

**Methods:**

150 CT and MRI datasets were initially read by two trained residents, and then supervised by two subspecialized radiologists. Recurrence rates by NI-RADS category were calculated, and receiver operating characteristic (ROC) curves were plotted. After dichotomization of the NI-RADS system (category 1 *vs* categories 2 + 3+4 and categories 1 + 2 *vs* 3 + 4), sensitivity, specificity, positive and negative predictive value were calculated.

**Results:**

26% of the reports were modified by the supervising radiologists. Area under the curve of ROC plots values of the supervision session were higher than those of the initial reading session for both the primary site (0.89 *vs* 0.86) and the neck (0.94 *vs* 0.91), but the difference was not statistically significant. For dichotomized NI-RADS category assignments, differences between the initial reading and the supervision session were statistically significant regarding specificity and PPV for the primary site (1 + 2 *vs* 3 + 4 and 1 *vs* 2 + 3+4) or even for both sites combined (1 *vs* 2 + 3+4).

**Conclusion:**

NI-RADS enables trained resident radiologists to report surveillance imaging in patients with treated oral squamous cell carcinoma with high discriminatory power. Additional supervision by a subspecialized head and neck radiologist particularly improves specificity of radiological reports.

## Introduction

The Neck Imaging Reporting and Data System (NI-RADS) is a standardized risk stratification tool for reporting surveillance imaging findings of patients who underwent curatively intended resection of head and neck (HN) cancer.^1,2^ The findings, as defined in a lexicon, determine numerical categories between 1 and 4 to be assigned separately for the primary site and the neck (cervical lymph nodes) with higher categories indicating a higher probability of cancer recurrence. In addition to providing a standardized framework for reporting radiological findings, NI-RADS links the assigned categories with recommendations for the patient’s further surveillance program. Originally designed for contrast-enhanced CT (CECT) with or without positron emission tomography (PET), NI-RADS can also be used to interpret contrast-enhanced magnetic resonance imaging (CEMRI).^1,3^

The implementation of a standardized reporting scheme such as NI-RADS in clinical routine has prerequisites both for the system itself and for the radiology department using it. First, a high discriminatory power of the reporting system’s categories is necessary to spare patients unnecessary examinations or procedures, on the one hand, and to prevent unnecessary burdens on the health-care system from redundant follow-up examinations, on the other. With regard to NI-RADS, any assignment of a category higher than 1 increases the number of follow-up examinations in the course of the patients’ surveillance programme. In particular, the correct differentiation between NI-RADS category 2 and 3 is crucial since a category 3 finding would recommend an invasive procedure (biopsy), whereas a category 2 finding would imply a non-invasive follow-up procedure (inspection or shortening of the surveillance interval).

Second, radiologists must be familiarized with the system and trained in its use in order to ensure high validity and reliability. Furthermore, the necessity of double reading should be evaluated. This includes the well-established concept of supervision, which means that the reports of residents in training are checked and discussed by subspecialized radiologists.^4,5^ However, a remaining question is what specific impact a supervision has on reports which are already prepared based on predefined terms and classifiers according to NI-RADS.

Therefore, the present study investigates how supervision by subspecialized head and neck radiologists influences discriminatory power of NI-RADS when initially utilized by radiologists in training. While NI-RADS is applicable to all types of HN cancers, the present study analyses CT and MRI surveillance of patients treated for oral squamous cell carcinoma (OSCC) since this is the most common entity and site.^6,7^

## Methods and materials

### Study design and patient selection

All data of this retrospective study were retrieved from an established database of our (Charité – Universitätsmedizin Berlin, corporate member of Freie Universität Berlin, Humboldt Universität zu Berlin, and Berlin Institute of Health, Campus Benjamin Franklin) institution comprising 525 contrast-enhanced CT and MRI datasets (in the following referred to as “imaging datasets”) acquired in 167 patients who underwent curatively intended resection of an OSCC and subsequently participated in our hospital’s interdisciplinary surveillance program.

Confirmation of diagnosis was available for all imaging datasets according to a reference standard for cancer recurrence. This was defined as either histopathological proof or unequivocal malignant findings in subsequent imaging and/or clinical surveillance examinations within 6 months of the first surveillance imaging examination. Imaging datasets without confirmed cancer recurrence according to these criteria were classified as recurrence-free. Imaging datasets of patients who were diagnosed with a secondary malignancy during the surveillance period or on subsequent imaging were excluded.

Finally, a total of 150 imaging datasets from different individuals, 50 with confirmed recurrence for the primary site or neck and 100 classified as disease-free, were included in this study. Each radiologist performing initial reading or supervision was randomly assigned 75 imaging datasets (Mersenne Twister Random Generator embedded in Microsoft Excel).

**Figure 1.**
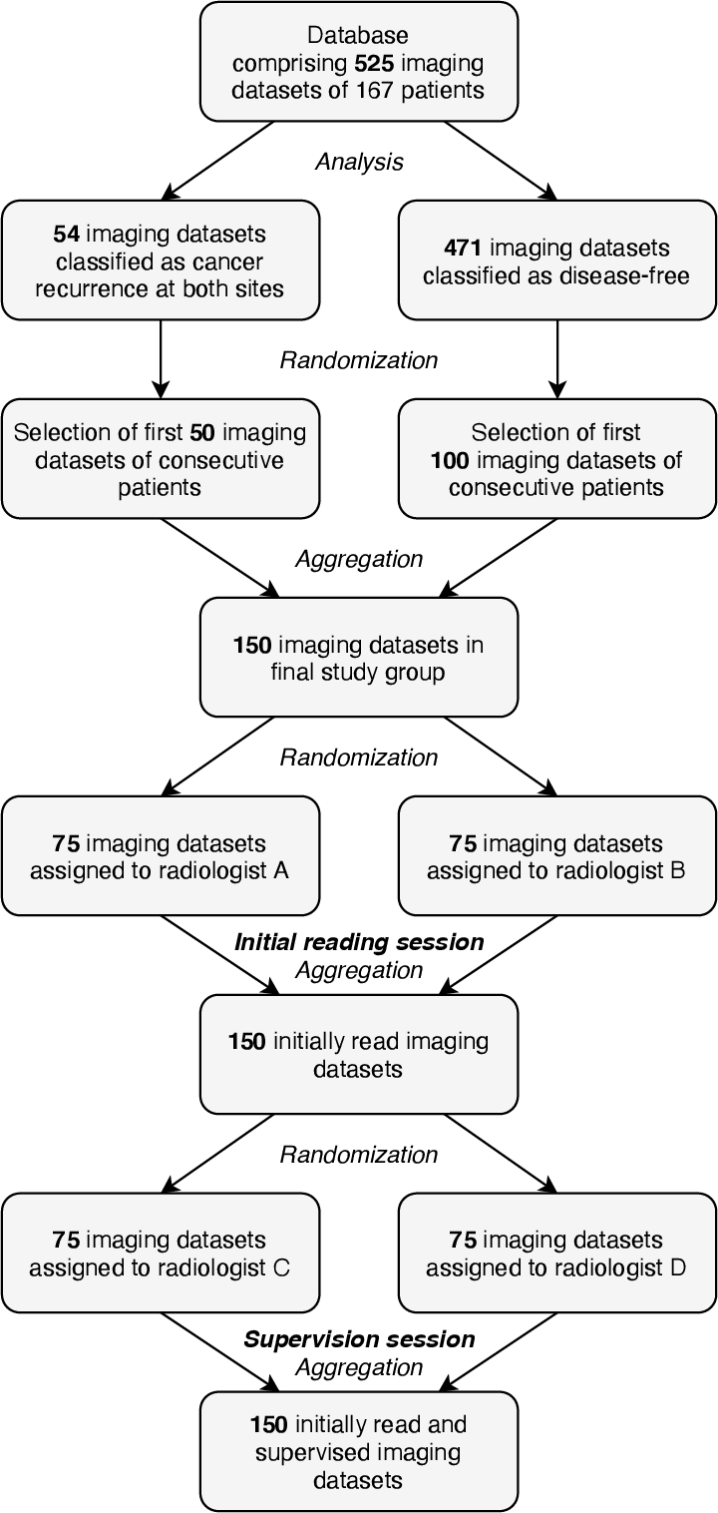
Flow of participant selection.

A flow of participant selection illustrating the above mentioned process is presented in Figure 1.

### Institutional surveillance program

The surveillance program follows the national guideline for the diagnosis and therapy of oral cavity carcinoma,^8^ which recommends clinical follow-up at 3 month intervals in the first 2 years and at 6 month intervals in the following 3 years. Overall, the surveillance program covers at least 5 years. Regarding imaging, CT and MRI are the modalities of choice whereas PET should only be used when cancer recurrence is suspected. The national guideline does not specify the time point of the first surveillance imaging; in our institution it takes place about 6 weeks after the completion of curatively intended therapy. Subsequently, further surveillance imaging should be performed every 6 months in the first 2 years and once a year in the following 3 years.

### Imaging datasets

Our study included both in- and outpatient CT and MRI examinations. Protocol requirements for CT datasets were: (1) small field of view (*i.e.* not larger than 250 × 250 mm), (2) arms positioned alongside the chest, (3) split-bolus injection of contrast medium to achieve a simultaneous arterial and venous phase, and (4) images acquired in axial, coronal and sagittal planes with a maximum reconstructed slice thickness of 3 mm. Protocol requirements for MRI datasets were: (1) small field of view (*i.e.* not larger than 250 × 250 mm), (2) axial *T*_1_ weighted images after contrast medium injection, and (3) axial *T*_2_ weighted fat-saturated images with a maximum slice thickness of 3 mm.

### Image interpretation

Imaging datasets were initially read by two resident radiologists (A and B), both with 5 years of experience, and then supervised by two radiologists (C and D) with 6 and 7 years of experience and subspecialization in head and neck imaging holding responsibility for the interdisciplinary tumor boards in collaboration with our hospital’s Department of Oral and Maxillofacial Surgery and Department of Otolaryngology. All contributing radiologists interpreted at least 200 HN imaging datasets using the NI-RADS scheme prior to this study. The radiologists had access to the patients’ medical records including reports from surgery and clinical examinations, laboratory data and information on pre-existing conditions reflecting the course of tumor surveillance until the interpreted imaging study was conducted. Radiologists performing the initial reading and supervision were also asked to rate the image quality of each CT or MRI dataset on a dichotomous scale with regard to interpretability in the presence of artifacts (sufficient/insufficient).

### Data analysis

Data analysis was performed using “RStudio” (RStudio, Boston, MA, v. 1.3.1093) including the packages listed below. Assignments of NI-RADS categories and cancer recurrences were counted, and cancer recurrence rates (positive predictive values, PPVs) were calculated for single and combined NI-RADS categories (1 + 2 *vs* 3 + 4, 1 *vs* 2 + 3+4). Receiver operating characteristic (ROC) curves were created using the “ROCR” package. The non-parametric DeLong test provided in the “pROC” package was used to compare areas under two correlated ROC curves (AUC).^9^ Alluvial plots were created using the “ggplot2” package.

Additionally, after dichotomizing NI-RADS category assignments (1 + 2 *vs* 3 + 4, 1 *vs* 2 + 3+4), sensitivities, specificities, negative predictive values (NPVs) and PPVs were calculated for the initial reading and supervision session and for the primary site, neck and both sites combined. Sensitivity and specificity were compared using the McNemar test,^10^ and generalized score statistics were calculated to compare PPVs and NPVs, both provided in the “DTComPair” package.^11^ Statistical significance was assumed for *p* < 0.05.

## Results

140 CT and 10 MRI data sets of 150 patients (65 female, 85 male; median age 62 years, range 38–92 years) obtained between April 2012 and April 2020 were included in this study. Participant characteristics including primary site, TNM (tumor, node, metastasis) category and information on adjuvant therapy are compiled in Table 1. 133 inpatient and 17 outpatient imaging datasets were included. The imaging datasets analysed in our study were acquired a median of 15 months (range 2–47 months) after the start of the surveillance program. There were no dropouts due to nondiagnostic image quality.

**Table 1.** Participant characteristics

**Primary site**	**n (%**)
Maxilla	12 (8%)
Mandible	30 (20%)
Palate	2 (1.3%)
Tongue	37 (24.7%)
Mouth floor	55 (36.7%)
Buccal mucosa	14 (9.3%)
**Primary category**	**n (%**)
pT1	57 (38%)
pT2	49 (32.7%)
pT3	11 (7.3%)
pT4a/pT4b/pT4c	33 (22%)
**Nodal category**	**n (%**)
pN0/cN0	86 (57.3%)
pN1	23 (15.3%)
pN2a/pN2b/pN2c	29 (19.3%)
pN3a/pN3b	12 (8%)
**Distant category**	**n (%**)
cM0	143 (95.3%)
pN1/cM1	7 (4.7%)
**UICC 8 Stage**	**n (%**)
I	46 (30.7%)
II	26 (17.3%)
III	19 (12.7%)
IVa/IVb/IVc	59 (39.3%)
**Adjuvant therapy**	**n (%**)
None	96 (64%)
Radiotherapy	20 (13.3%)
Chemotherapy	3 (2%)
Radiochemotherapy	31 (20.7%)

Table 2 (primary site), Table 3 (neck) and Table 4 (both sites combined) list the counts of assignments by NI-RADS category and numbers of those of cancer recurrences with calculated recurrence rates, all separately for initial reading and supervision sessions. Recurrence rates gradually increased with higher NI-RADS categories except for category 2 of the primary site in the supervision session, where both imaging datasets classified as category 2b were confirmed as cancer recurrence.

**Table 2.** Category, recurrence and recurrence rate counts for the primary site

NI-RADS category	Initial reading session			Supervision session		
	Category count	Recurrence count	Recurrence rate (PPV)	Category count	Recurrence count	Recurrence rate (PPV)
1	112	8	7.1%	121	7	5.8%
2a	6	1	16.7%	0	0	0%
2b	3	2	66.7%	2	2	100%
2 (2*a* + 2b)	9	3	33.3%	2	2	100%
3	12	7	58.3%	12	10	88.9%
4	17	16	94.1%	15	15	100%
2 + 3 + 4	38	26	68.4%	29	27	93.1%
1 + 2	121	11	9.1%	123	9	7.3%
3 + 4	29	23	79.3%	27	25	92.6%

PPV, Positive predictive value.

**Table 3.** Category, recurrence and recurrence rate counts for the neck

NI-RADS category	Initial reading session			Supervision session		
	Category count	Recurrence count	Recurrence rate (PPV)	Category count	Recurrence count	Recurrence rate (PPV)
1	116	5	4.3%	113	3	2.7%
2	10	2	20%	10	1	10%
3	11	10	90.9%	16	15	93.8%
4	13	13	100%	11	11	100%
2 + 3 + 4	34	25	73.5%	37	27	73.0%
1 + 2	126	7	5.6%	123	4	3.3%
3 + 4	24	23	95.8%	27	26	96.3%

PPV, Positive predictive value.

**Table 4.** Category, recurrence and recurrence rate counts for the primary site and neck combined

NI-RADS category	Initial reading session			Supervision Session		
	Category count	Recurrence count	Recurrence rate (PPV)	Category count	Recurrence count	Recurrence rate (PPV)
1	228	13	5.7%	234	10	4.3%
2	19	5	26.3%	12	3	25%
3	23	17	73.9%	28	25	89.3%
4	30	29	96.7%	26	26	100%
2 + 3 + 4	72	51	70.8%	66	54	81.8%
1 + 2	247	18	7.3%	246	13	5.3%
3 + 4	53	46	86.8%	54	51	94.4%

PPV, Positive predictive value.

**Figure 2.**
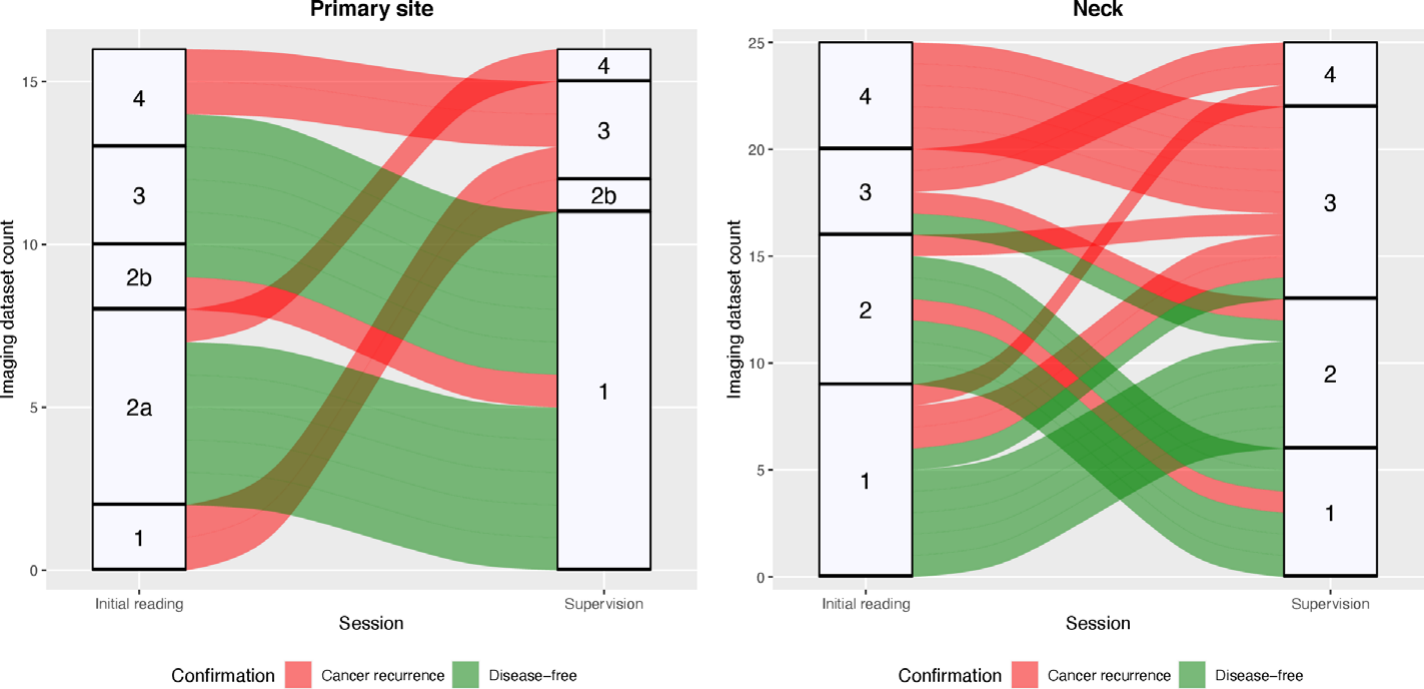
Alluvial plots illustrating the imaging dataset-specific changes in NI-RADS category assignment for the primary site (left) and the neck (right) between the initial reading and supervision session. Flows are color-coded according to the results of the confirmation studies.

**Figure 3.**
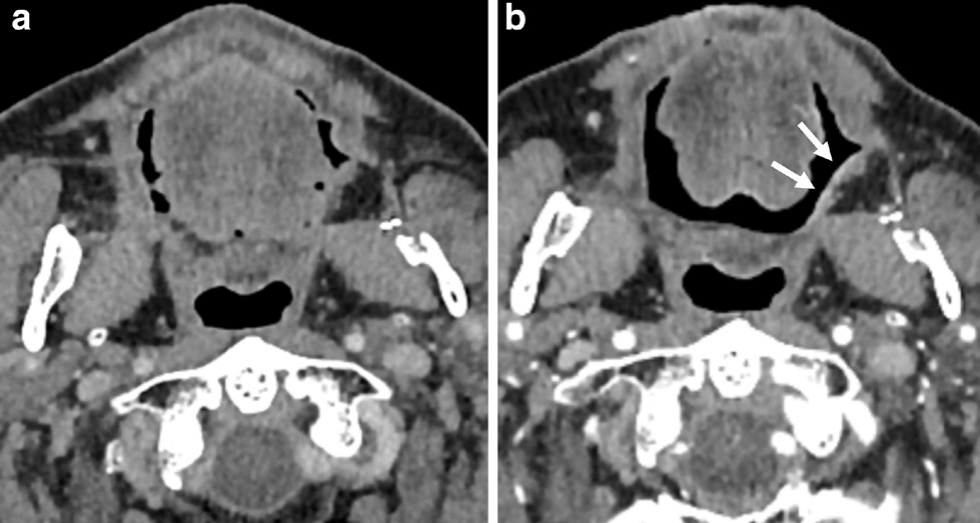
a,b. Post-treatment surveillance CT images of a patient with OSCC located primarily in the left mouth floor obtained 18 months (**a**) and 24 months (**b**) after resection and adjuvant radiotherapy. There is new enhancement on the surface of the radialis flap reconstruction (b, indicated by white arrows). The initial assignment of NI-RADS category 2a for the primary site was downgraded to category 1 by the supervising radiologist. There was no evidence of cancer recurrence in clinical inspection and subsequent surveillance CT. OSCC, oral squamous cell carcinoma

**Figure 4.**
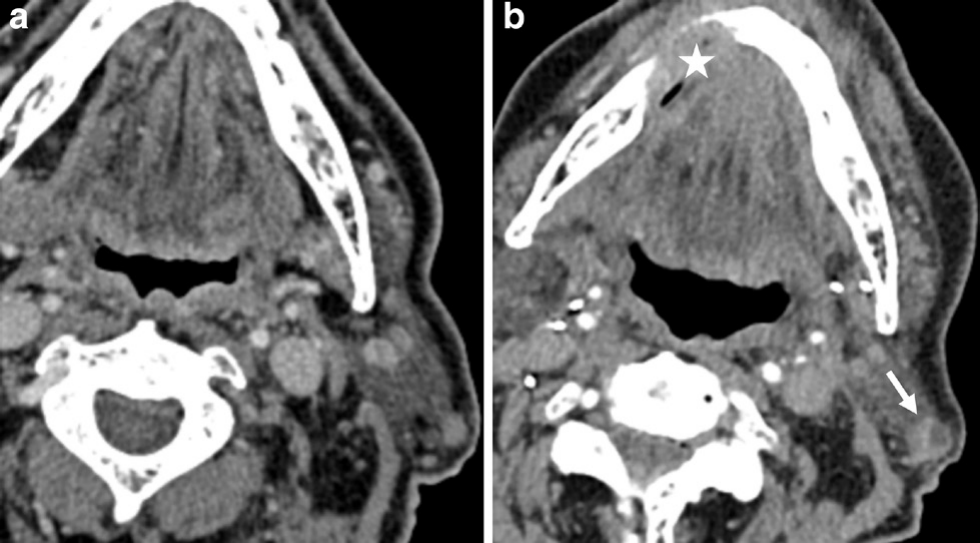
a,b. Post-treatment surveillance CT images of a patient with OSCC located in the right mandible obtained 3 months (**a**) and 6 months (**b**) after resection. A parotid lymph node on the right side-shows new focal necrosis (b, indicated by white arrow). The initial assignment of NI-RADS category 1 for the neck was upgraded to category 3 by the supervising radiologists. Histopathology confirmed cancer recurrence, also for the primary site (indicated by white star). OSCC, oral squamous cell carcinoma.

Supervising radiologists C and D made changes to a total of 39 (26%) NI-RADS reports from radiologists A and B who performed the initial reading. In this process, 16 (10.67%) NI-RADS ratings for the primary site and 25 (16.67%) ratings for the neck were modified. Alluvial plots in Figure 2a (primary site) and Figure 2b (neck) illustrate the flow of category modifications with respect to the results of the confirmation studies, and example cases are shown in Figures 3 and 4. Correspondingly, 111 reports (74%) went through the supervision process without any changes to the initially assigned NI-RADS categories.

**Figure 5.**
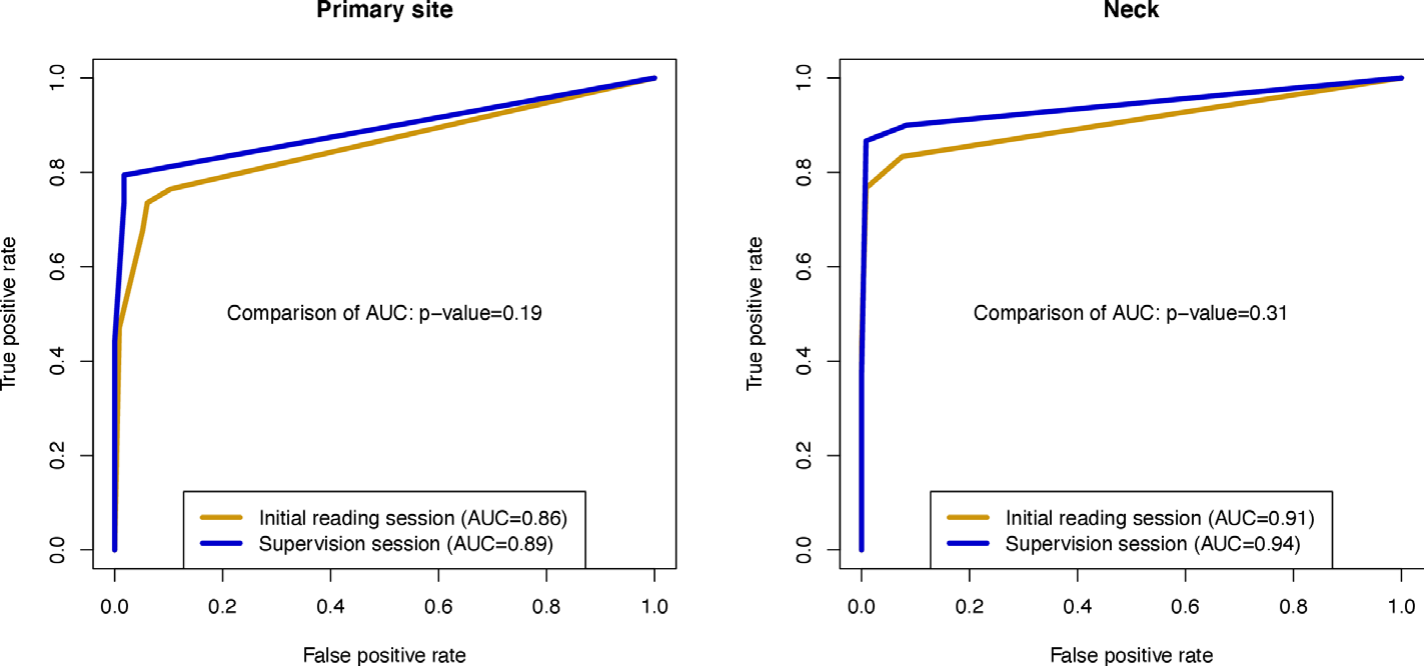
ROC curves comparing the performance of NI-RADS in cancer recurrence detection for the primary site (left) and neck (right) between the initial reading and supervision session. AUC, area under the curve

AUC values for ROC plots of the supervision session were higher than those of the initial reading session for both the primary site (Figure 5a) and the neck (Figure 5b), but the difference was not statistically significant.

Analysis of dichotomized NI-RADS category assignments showed a statistically significant difference between the initial reading and the supervision session in specificity and PPV for the primary site (1 + 2 *vs* 3 + 4, 1 *vs* 2 + 3 + 4) or even for both sites combined (1 *vs* 2 + 3 + 4) (Tables 5 and 6).

**Table 5.** Sensitivity, specificity and predictive values after dichotomizing NI-RADS category assignment (1 + 2 vs 3 + 4)

	Sensitivity	Specificity	PPV	NPV
Primary site				
Initial reading session	67.6	94.8	79.3	90.9
Supervision session	73.5	98.3	92.6	92.7
*p*	.16	.04	.03	.1
Neck				
Initial reading session	76.7	99.2	95.8	94.4
Supervision session	86.7	99.2	96.3	96.7
p	.18	>.99	.93	.18
Both sites combined				
Initial reading session	71.9	97.0	86.8	92.7
Supervision session	79.7	98.7	94.4	94.7
*p*	.07	.1	.18	.93

NPV, Negative predictive value; PPV, Positive predictive value.

**Table 6.** Sensitivity, specificity and predictive values after dichotomizing NI-RADS category assignment (1 *vs* 2 + 3+4)

	Sensitivity	Specificity	PPV	NPV
Primary site				
Initial reading session	76.5	89.7	68.4	92.9
Supervision session	79.4	98.3	93.1	94.2
*p*	0.56	<0.01	<0.01	0.34
Neck				
Initial reading session	83.3	92.5	73.5	95.7
Supervision session	90.0	91.7	73.0	97.3
*p*	0.32	0.76	0.94	0.32
Both sites combined				
Initial reading session	79.7	91.1	70.8	94.3
Supervision session	84.4	94.9	81.8	95.7
*p*	0.26	0.04	0.03	0.19

NPV, Negative predictive value; PPV, Positive predictive value.

## Discussion

Modification of initial reports by supervising HN radiologists slightly improved nearly all diagnostical performance metrics of NI-RADS (*e.g.* AUC values: from 0.86 to 0.89 for the primary site and from 0.91 to 0.94 for the neck), and differences in specificity and PPV for the primary site (1 + 2 *vs* 3 + 4 and 1 *vs* 2 + 3+4+) or even both sites (1 *vs* 2 + 3+4) after dichotomization of the NI-RADS system were statistically significant. The main findings of the present study are twofold. First, the results suggest that NI-RADS and its accompanying lexicon enable trained radiology residents to accurately report surveillance imaging of patients treated for HN malignancy already without supervision by subspecialized radiologists. Data on how double reading affects the diagnostic accuracy of standardized reports of HN cross-sectional imaging findings are sparse, and thus direct comparison of our results with other studies is not possible. However, there is growing evidence from other fields that training residents using the respective standard reporting system leads to higher diagnostic accuracy and thus a lower rate of report recalls. In a study of breast ultrasonography using BI-RADS, Yoon et al^12^ showed that diagnostic accuracy of 61 residents improved after training on the lexicon and reached similar levels as those achieved by subspecialized radiologists although the latter group remained ahead in terms of overall accuracy. Similar observations have been reported for PI-RADS. Rosenkrantz et al^13^ found that a group of six novice radiologists showed a distinct improvement in diagnostic accuracy followed by a plateau phase after initially familiarizing themselves with the examination itself as well as with the reporting system in the process of reading 40 prostate MRI scans meant for training purposes. Furthermore, Rosenkrantz et al^14^ concluded that initial improvement in diagnostic accuracy was largely driven by self-directed learning, whereas feedback from subspecialized radiologists rather had a longer-term impact on the sensitivity and diagnostic confidence of resident readers. While the first part of these conclusions is consistent with the results presented here, our study design does not allow for differentiating between the influence of self-directed learning and supervision on diagnostic accuracy. Of note, similar considerations as in the reported studies and in our study influenced the recent ESUR/ESUI consensus statement on multiparametric MRI for the detection of clinically significant prostate cancer, which describes supervised education and targeted training of residents on PI-RADS as an essential part of quality assurance.^14^

Second, although a high degree of accuracy is already achieved by trained residents, subspecialty radiologists achieve a higher diagnostic discriminatory power when using NI-RADS. For the primary site, the alluvial plot conspicuously shows a frequent downgrading to NI-RADS Category 1, for which the greater experience of the supervising radiologists may be decisive. A case to illustrate this kind of decision is presented in Figure 3. This tendency is also reflected in the significantly higher specificity after supervision for the primary site and both sites combined. Given these results, supervision of standardized HN reports has the potential to contribute to further economization of surveillance and reduces the need for more frequent and more invasive patient surveillance after treatment for OSCC. This finding is similar to comparable studies that investigated other standard reporting systems. A study of Luzzago et al^15^ found that a second reading of prostate MRI initially read by less-trained radiologists led to a change in clinical management in about half of all patients and approximately, a third of males could avoid prostate sampling. The share of recalled reports and thus the percentage of cases with potential changes in clinical management in the study of Luzzago et al^15^ is significantly higher than in our study. An important difference might be that the initial readers in our study already had a certain level of experience with NI-RADS. The alluvial plots show that results for both initial and supervision ratings were more inconsistent for the neck than for the primary site. This may be related to a more general issue in radiology regarding the grading of potential lymph node involvement since there is no consensus on malignancy criteria yet although many criteria have been proposed.^16,17^ Regarding NI-RADS, this issue could be mitigated by specifying ategory 2 for the neck on the basis of a metric threshold for size progression, as proposed in the recently published Node-RADS.^18^ Given the lack of such consensus criteria in the current version of NI-RADS, the case presented in Figure 4 illustrates what role supervision may have despite the high diagnostic accuracy we found for the initial readers.

Although we performed a granular analysis of the influence of double reading on NI-RADS reports, there are some limitations to be mentioned. First, the gap of experience in terms of work years between the residents and the supervising radiologists is small. The difference in experience relevant to this study results in particular from the subspecialization in the field of HN radiology over several years. Second, this study does not account for the influence of supervision on reports by novice radiologist as both initial readers in our study already had several years of experience. Such a comparison would likely yield a stronger discrepancy between initial reading and supervision. Third, our study design only allows measurement of the diagnostic accuracy of initial reading combined with supervision and not of the accuracy of the reading of the subspecialized radiologists alone. Therefore, it cannot be ruled out that the initial reading influenced the rating of the subspecialized radiologist to a certain degree, but nevertheless the study design was chosen to reflect the clinical routine accurately. Lastly, we predominantly included CT datasets with only a very small share of 10 MRI datasets (6.7%), which is also why no subgroup analysis was conducted. On the other hand, this proportion reflects the importance of CT as the preferred modality in imaging surveillance of HN cancer in many institutions based on availability and economic considerations.

## Conclusions

NI-RADS enables trained resident radiologists to report the findings of surveillance imaging of patients with oral squamous cell carcinoma with high discriminatory power. Nevertheless, additional supervision by a subspecialized head and neck radiologist is of particularly high importance for evaluation of the primary site and reduces the need for more invasive and more frequent surveillance.
